# Conservative Treatment with Teriparatide Versus Vertebroplasty for Acute Osteoporotic Vertebral Compression Fractures: A Meta-Analysis

**DOI:** 10.3390/jcm14113967

**Published:** 2025-06-04

**Authors:** Subum Lee, Junseok W. Hur, Younggyu Oh, Sungjae An, Yeongu Chung, Danbi Park, Jin Hoon Park

**Affiliations:** 1 Department of Neurosurgery, Korea University Anam Hospital, Korea University College of Medicine, Seoul 02841, Republic of Korea; lee_s@korea.ac.kr (S.L.); hurjune@gmail.com (J.W.H.); oyk1223@hanmail.net (Y.O.); annuguri88@gmail.com (S.A.); 2 Department of Neurosurgery, Kangbuk Samsung Hospital, Sungkyunkwan University School of Medicine, Seoul 03181, Republic of Korea; yeongu.chung@gmail.com; 3 Department of Neurological Surgery, Asan Medical Center, University of Ulsan College of Medicine, 88 Olympic-ro 43-gil, Songpa-gu, Seoul 05505, Republic of Korea; danbi08@kakao.com

**Keywords:** compression fracture, conservative treatment, meta-analysis, osteoporotic fracture, teriparatide, vertebroplasty

## Abstract

**Background/Objectives:** The debate continues, despite numerous studies, on whether vertebroplasty (VP) or conservative treatment is more suitable for osteoporotic vertebral compression fractures (OVCFs). Meanwhile, teriparatide (TP) has shown promise in accelerating bone healing in OVCFs. This analysis aims to clarify the potential benefits of conservative treatment using TP over VP from several clinical studies on acute OVCFs. **Methods:** A literature search was performed, using the MEDLINE, Embase, Cochrane Review, Web of Science, and Google Scholar databases, for studies published up until September 2023. Five studies [one randomized controlled study (RCT) and four non-RCTs] were included in a qualitative and quantitative synthesis. Data were extracted and analyzed using a random-effects model to obtain the effect size. **Results:** Five studies with a total of 326 (TP = 147, VP = 179) patients were included. Within the first week of treatment, the VP group showed a significantly greater decrease in their visual analog scale (VAS) scores. There was no significant difference in VAS score reduction between the two groups from one to three months. However, after 6 months, the TP group exhibited significant superiority in VAS scores and bone mineral density (BMD). Furthermore, TP was associated with a reduced number of new-onset OVCFs, with a statistically significant estimated odds ratio of 0.15 (95% CI, 0.04–0.51, *p* < 0.01). **Conclusions:** Conservative treatment using TP for acute OVCF has been found to reduce subsequent fractures, provide equivalent or superior pain control, and increase BMD compared to VP. Nonetheless, the meta-analysis results are weak, due to the low level of evidence.

## 1. Introduction

Patients with symptomatic osteoporotic vertebral compression fractures (OVCFs) often experience severe back pain, significantly impacting their health-related quality of life [1,2]. Various treatment strategies for OVCFs exist, but they remain controversial [2,3]. Conventional conservative treatment options for symptomatic OVCFs include narcotic analgesics, bed rest, and bracing [4,5,6]. Moreover, physical therapy interventions including postural correction, core strengthening, and functional rehabilitation are often employed to relieve pain and restore mobility in these patients, serving as essential components of conservative care [7].

However, the majority of OVCF patients undergoing conventional conservative treatments experience prolonged pain and a reduced quality of life [8]. Persistent back pain beyond the acute phase is frequently reported in OVCF patients, and may result from vertebral deformity, kyphosis, spinal instability, or nonunion [9]. Progressive vertebral collapse and failure of fracture healing are not uncommon following conservative treatment [10], potentially necessitating surgical interventions like percutaneous vertebroplasty (VP).

Over the past decades, VP has been extensively used worldwide to treat painful OVCFs [1,11]. The literature reports that VP effectively alleviates acute pain and restores patients’ walking ability post-procedure, especially when compared with conservative care [11,12]. However, high-quality, double-blinded, randomized controlled trials (RCTs) have not shown any significant difference in pain reduction between VP and sham procedures [13,14,15]. Based on the evidence, the Cochrane review also does not endorse the use of VP for treating acute or subacute OVCFs in routine practice [16].

The primary goal in treating OVCFs is to ensure fracture healing and prevent secondary OVCFs. Conceptually, recombinant human parathyroid hormone (teriparatide, TP) may accelerate fracture healing during the acute stage of OVCFs [17,18,19,20]. Additionally, TP itself might have a pain-reducing effect due to its inhibitory impact on inflammatory cytokine expression [21]. However, the clinical efficacy of TP in the conservative treatment of acute OVCF remains unclear.

The debate over the effectiveness of traditional conservative treatment versus VP continues. The purpose of this study is to explore beyond this debate, investigating whether a novel conservative treatment based on a bone anabolic agent, TP, can be more effective than VP. Accordingly, we conducted a systematic review and meta-analysis by synthesizing published articles on this topic to clarify the clinical benefits of conservative treatment using TP and further investigate its potential as an alternative to VP for acute OVCF management.

## 2. Methods

This study is registered in PROSPERO (ID: CRD42024615450) and reported in accordance with the PRISMA (Preferred Reporting Items for Systematic Reviews and Meta-analyses) guidelines [22]. We conducted a meta-analysis and systematic review of clinical studies on conservative treatment with TP or percutaneous VP for acute OVCFs.

### 2.1. Search Strategy and Study Extraction

The MEDLINE (PubMed), Embase, Cochrane Review, and Web of Science databases, and the title field of Google Scholar, were searched for articles published up until September 2023. The search queries included OVCF and TP or percutaneous VP synonyms and related terms, as follows: (“osteoporotic compression fracture” OR “vertebral compression fracture”) AND (“teriparatide” OR “parathyroid hormone”) AND (“vertebroplasty” OR “cement augmentation”). The search was not restricted to RCTs, and was extended to original articles, including non-RCTs. The decision for an article’s selection was primarily based on its title and a review of the abstract, followed by full-text screening. The two reviewers independently performed the study screening and data extraction, and any discrepancies were resolved by discussion between the two reviewers or with the entire research group.

The inclusion criteria were as follows: (1) studies involving patients with acute OVCFs; (2) comparative studies evaluating conservative treatment using TP versus percutaneous VP; (3) studies reporting relevant clinical outcomes, such as visual analog scale (VAS) scores for pain, bone mineral density (BMD), radiologic parameters, and complications; and (4) published original research articles, including RCT and non-RCTs. The exclusion criteria included the following: (1) non-comparative studies without direct comparisons between TP and VP; (2) animal studies, case reports, reviews, letters, and commentaries; (3) studies lacking sufficient or complete data as required for quantitative analysis; and (4) studies involving kyphoplasty. To minimize procedural heterogeneity, studies involving kyphoplasty were excluded. Although kyphoplasty and VP are both vertebral augmentation techniques, they differ in key aspects such as balloon inflation, cement volume, technique, and cost. Including both procedures could have introduced significant clinical variability; therefore, only VP was selected as a comparator to TP in this analysis. The studies that met the inclusion criteria and were ultimately analyzed were published between 2013 and 2022, as described in the Section 3.

### 2.2. Data Extraction and Quality Assessment

Reference data for each treatment method group (TP and VP groups), comparative results of the clinical outcomes (VAS score for back pain), radiologic outcomes [T-score of BMD, kyphotic angle (KA) at fracture level, anterior height (AH) loss, or compression ratio change at fracture level], and adverse event occurrence (new-onset OVCF) were extracted from the selected articles (Table 1). The dichotomous variable included adverse events, specifically new-onset OVCFs, which were extracted to estimate the odds ratio. Continuous variables such as the mean and standard deviation of VAS scores were extracted to estimate mean differences. The risk of bias was assessed by two authors independently with the ROBINS-I (Risk Of Bias In Non-randomised Studies–of Interventions) tool for observational studies and the RoB-2 (Risk of Bias for randomized trials) tool for RCTs [23,24]. Disagreements were resolved by discussion between 2 reviewers or with the entire research group.

### 2.3. Statistical Analysis

R software was used to conduct statistical analysis (ver. 4.3.1; The R Foundation for Statistical Computing, Vienna, Austria). Tests of heterogeneity were performed using $I^{2}$ statistics. The interpretation of $I^{2}$ was categorized according to its range, as follows: 0–25% indicates low heterogeneity, 25–50% suggests moderate heterogeneity, 50–75% represents high heterogeneity, and 75–100% signifies very high heterogeneity. The parameter with $I^{2}$ values of *p* < 0.05, which was considered to have a significantly high degree of heterogeneity, was additionally validated by subgroup analysis or sensitivity analysis. A random-effects model was applied to obtain the effect size and its statistical significance, because it was assumed that the subjects and methods of the included studies performed by independent researchers could not be entirely equivalent and, therefore, could not have a common effect size. A probability of *p* < 0.05 was considered statistically significant. The results were expressed as the mean difference and 95% CI for continuous outcome data, and in the form of an odds ratio and 95% CI for dichotomous outcome data.

## 3. Results

### 3.1. Literature Search

**Figure 1:**
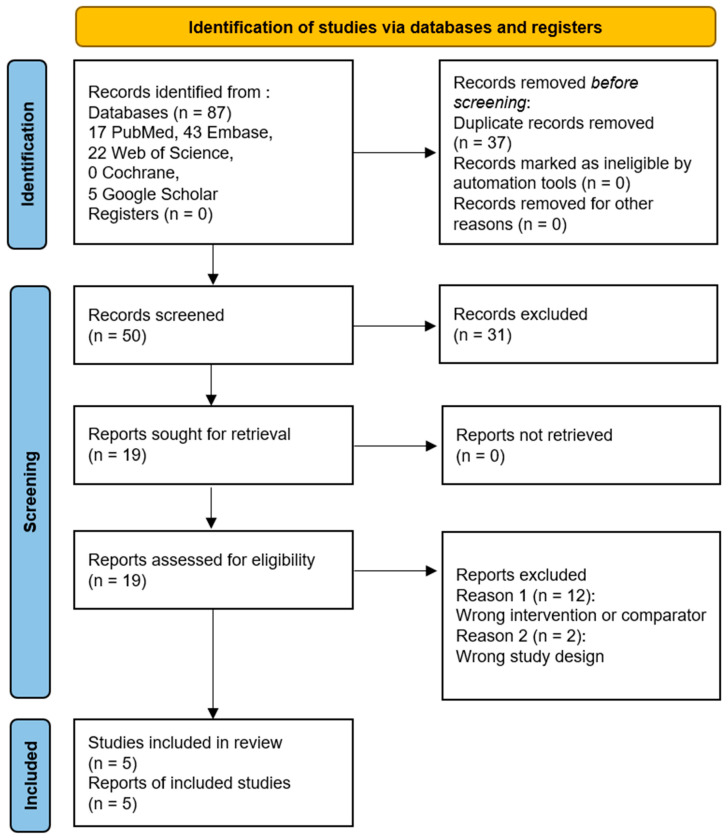
PRISMA flow chart diagram [22].

A preliminary literature search utilizing the subject headings revealed 17 articles from PubMed, 43 studies from Embase, 22 studies from Web of Science, 0 studies from the Cochrane clinical trials database, and 5 studies from the title field of Google Scholar Advanced search. There were 37 duplicates among these 87 studies; thus, they were eliminated. After evaluating the remaining 50 titles and abstracts, those that included content regarding the clinical outcomes after TP or VP and provided comparative results between the two treatment methods were finally selected. Finally, a total of five studies were included in the meta-analysis (Table 1). Figure 1 presents the detailed selection process.

### 3.2. Quality Assessment

**Figure 2:**
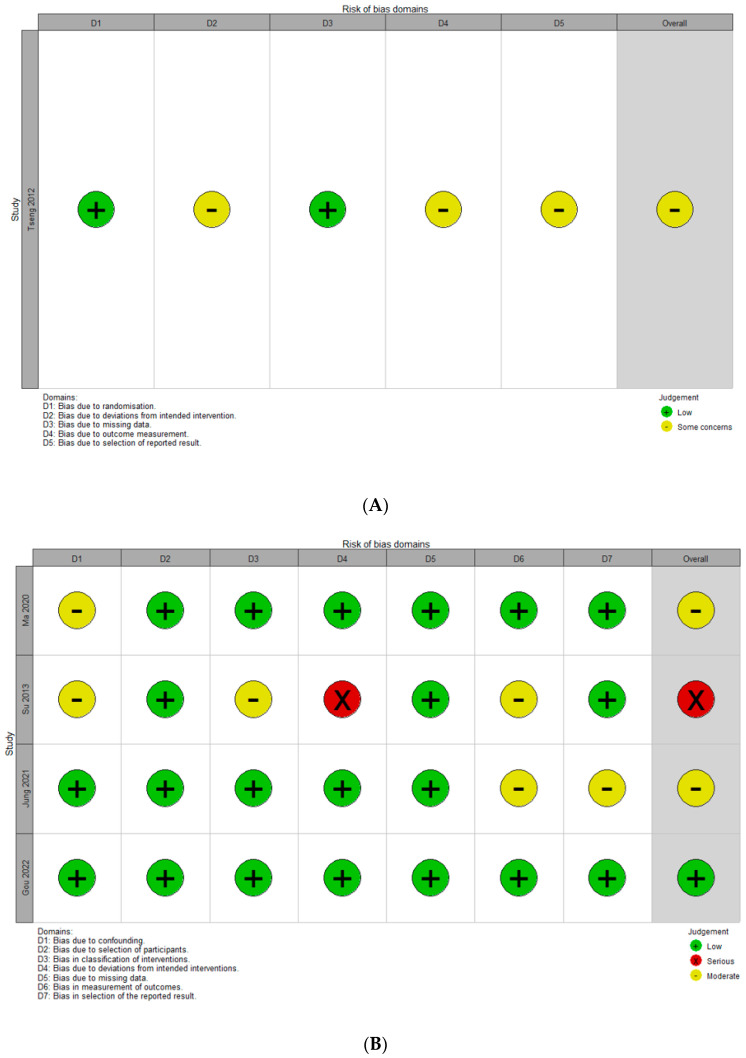
Quality assessment for extracted studies. (**A**) Risk of bias assessment using the RoB-2 (a revised tool to assess risk of bias in randomized trials) for a randomized controlled study [25]. (**B**) Risk of bias assessment using the ROBINS-I (Risk of Bias in Non-randomized Studies of Interventions) tool [10,26,27,28].

The overall quality assessment using the five domains of the RoB-2 tool for RCTs and the seven domains of the ROBINS-I tool for non-RCTs is shown in Figure 2. One RCT study was assessed as presenting some concerns regarding the domains of deviations from intended intervention, outcome measurement, and selection of reported results, because the participants and researchers were aware of what the intervention was, and the deviations did not affect the outcomes. The mediator outcome evaluators were informed about the content of the intervention. Additionally, it was not specified whether the data analysis was conducted according to the pre-specified plan. Of the four non-RCTs, one study was rated as having a serious risk of deviation from the intended interventions. This is because imbalances between intervention groups that could affect the outcomes were found in the baseline patient age and BMD categories. Of the 28 domains across all studies, 21 domains (75.0%) were determined as low-risk; thus, the overall ROB was considered to be low. To enhance transparency, we added tabulated domain-level justifications for studies with a moderate or serious risk (Table 2).

### 3.3. VAS at Immediate Period (<1 Week)

**Figure 3:**
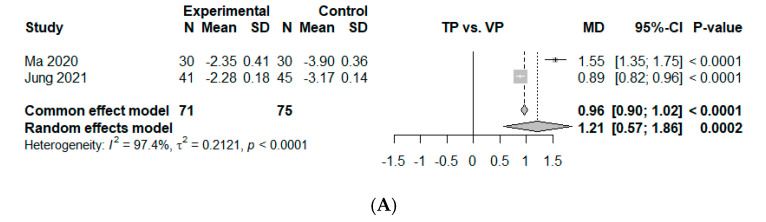

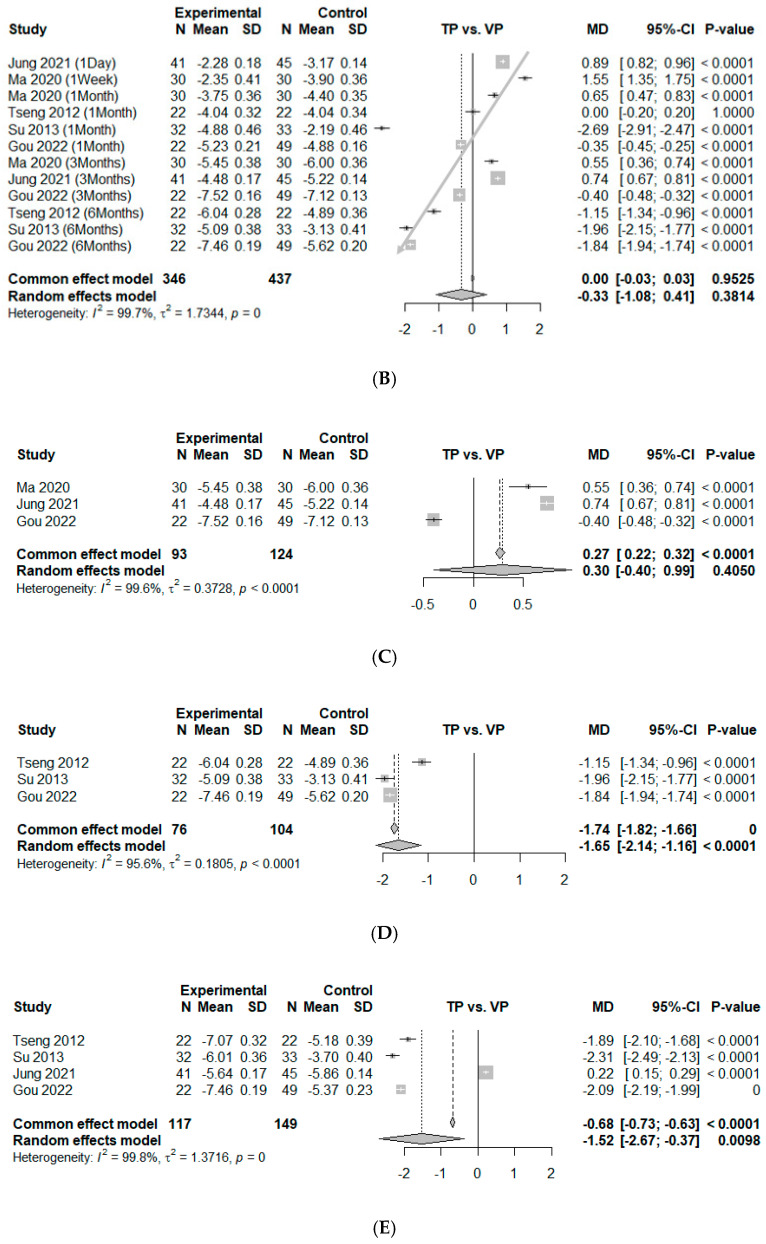
Forest plot of comparison [10,25,26,27,28]. (**A**) Mean difference for visual analog scale (VAS) change between baseline and 1 week. (**B**) Mean difference for visual analog scale (VAS) change between baseline and 6 months. Studies are arranged according to follow-up period, ranging from immediately after treatment to 6 months. (**C**) Mean difference for VAS change between baseline and 3 months. (**D**) Mean difference for VAS change between baseline and 6 months. (**E**) Mean difference for VAS change between baseline and 12 months. Experimental, conservative treatment using teriparatide; Control, vertebroplasty; MD, absolute mean difference (weighted mean difference); SD, standard deviation; standardized mean difference; CI, confidence interval.

Two comparisons from five studies presented continuous data on VAS improvement at an immediate time point within a week. The estimated overall mean difference was 1.21 (95% CI, 0.57 to 1.86, *p* < 0.01), indicating significant superiority of VP over TP (Figure 3A). Significant heterogeneity was observed ($I^{2}$ = 97.4%, *p* < 0.01).

### 3.4. VAS at 0–6 Months

Twelve comparisons from five studies presented continuous data on VAS improvement at 0–6 months, which could be used for an analysis of effect size by mean difference [10,25,26,27,28]. The data showed no significance between TP and VP, with an estimated mean difference of −0.33 (95% CI, −1.08 to 0.41, *p* = 0.38). Significant heterogeneity was found ($I^{2}$ = 99.7%, *p* < 0.01). During this period, there is a consistent trend where the point estimates for VAS reduction shift more favorably from VP to TP as time progresses (the studies were arranged according to the follow-up period). We represent this time-dependent trend using gray arrows in Figure 3B, not for statistical validation, but to illustrate the observed pattern over time (Figure 3B). Since these time points represent different clinical contexts, we divided the data into two separate analyses, focusing on the 3-month and 6-month intervals, while excluding data from other time points during this period. At the 3-month mark, no significant difference in pain between the two groups was observed, with an estimated mean difference of 0.30 (95% CI, −0.40 to 0.99, *p* = 0.41). Significant heterogeneity was noted ($I^{2}$ = 99.6%, *p* < 0.01) (Figure 3C). However, at the 6-month mark, a significant difference in pain reduction between the two groups was detected, with an estimated mean difference of −1.65 (95% CI, −2.14 to −1.16, *p* < 0.01). Significant heterogeneity was noted ($I^{2}$ = 95.6%, *p* < 0.01) (Figure 3D).

### 3.5. VAS at 12 Months

Four comparisons from four studies were included in an analysis of effect size by mean difference for VAS improvement at 12 months [10,25,27,28]. The study by Ma et al. was excluded, as it did not meet the 12-month follow-up requirement [26]. The estimated overall mean difference was calculated as −1.52 (95% CI, −2.67 to −0.37, *p* < 0.01), which meant significant superiority of TP over VP. The degree of heterogeneity was significantly high ($I^{2}$ = 99.8%, *p* = 0.00) (Figure 3E).

### 3.6. BMD Differences at 12 Months

**Figure 4:**
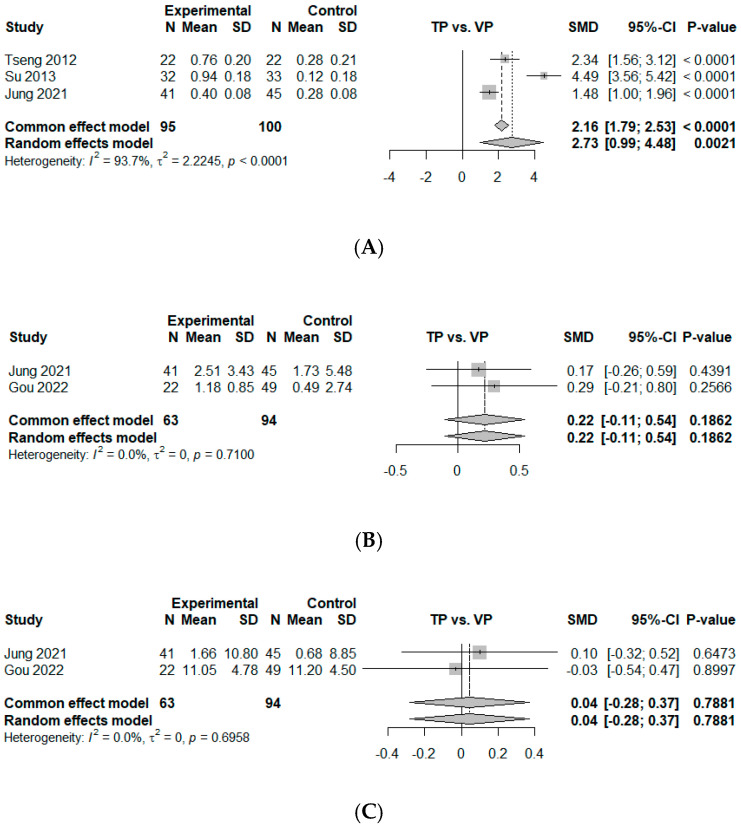

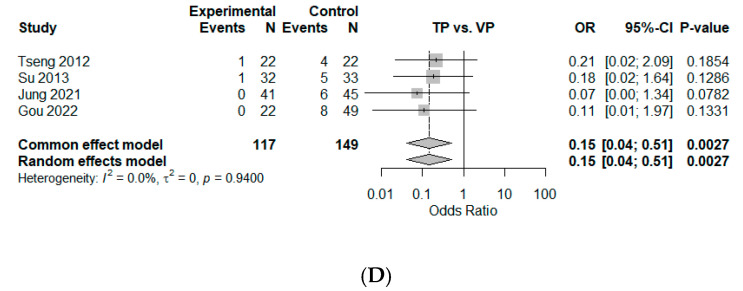
Forest plot of comparison [10,25,27,28]. (**A**) Mean difference for T-score of bone mineral density (BMD) change between baseline and 12 months. (**B**) Mean difference for kyphotic angle (KA, °) change between baseline and 12 months. (**C**) Mean difference for compression ratio based on average height of upper and lower adjacent vertebral bodies (%, Jung et al. [28]) or anterior height loss (%, Gou et al. [10]) between baseline and 12 months. (**D**) Odds ratio of new-onset subsequent osteoporotic vertebral compression fractures (OVCFs). Experimental, conservative treatment using teriparatide; Control, vertebroplasty; Events, new-onset OVCF; SD, standard deviation; SMD, standardized mean difference; CI, confidence interval.

Three comparisons from three studies were included in the measurement of effect size by mean difference for BMD improvement at 12 months, based on the mean difference between the TP and VP groups [25,27,28]. All BMD measurements included in the analysis were obtained using dual-energy X-ray absorptiometry (DEXA) from at least two evaluable vertebrae in the lumbar spine region (L1–L4). Additional details regarding the sources of BMD in each study are summarized in Table 3. The study by Gou et al. was excluded because it measured the Hounsfield units in computer tomography images [10], making it incomparable with other studies investigating BMD T-scores. The study by Ma et al. was also excluded because it only reported baseline BMD values without follow-up data [26]. The overall mean difference was estimated as 2.73 (95% CI, 0.99–4.48, *p* < 0.01), indicating that TP led to a significantly greater improvement in BMD compared to VP. Notably, all included studies consistently reported superior BMD gains for TP. A high degree of heterogeneity was revealed ($I^{2}$ = 93.7%, *p* < 0.01) (Figure 4A).

### 3.7. KA Differences at 12 Months

Two studies were included in the analysis of effect size by mean difference for KA at 12 months [10,28]. The estimated overall mean difference was calculated as 0.22 (95% CI, −0.11–0.54, *p* = 0.19), slightly favoring VP, without statistical significance. No heterogeneity was observed ($I^{2}$ = 0%, *p* = 0.71) (Figure 4B).

### 3.8. AH Loss or Compression Ratio Change at 12 Months

Two studies were included in the analysis of effect size by mean difference for the compression ratio or AH loss at 12 months [10,28]. The data showed no significant difference between TP and VP with an estimated mean difference of 0.04 (95% CI, −0.28–0.37, *p* = 0.79). No heterogeneity was observed ($I^{2}$ = 0%, *p* = 0.70) (Figure 4C).

### 3.9. New-Onset OVCFs

Four studies provided dichotomous data which could be included in the measurement of effect size by odds ratio for new-onset OVCFs [10,25,27,28]. This suggested that a smaller number of new-onset OVCFs was observed for TP than for VP, with an estimated odds ratio of 0.15 (95% CI, 0.04–0.51, *p* < 0.01), with statistical significance. No heterogeneity was observed ($I^{2}$ = 0%, *p* = 0.94) (Figure 4D).

### 3.10. Heterogeneity Handling

To investigate the substantial heterogeneity observed across studies, we conducted time point-specific and subgroup analyses for the differences in pain scores between the TP and VP groups at 3, 6, and 12 months. The overall heterogeneity was high at all time points.

To identify potential sources of heterogeneity, we performed subgroup analyses based on age (<75 vs. ≥75 years), fracture type (adjacent vs. new), co-medication (use of denosumab with TP or BP with VP), and study design (RCT vs. non-RCT) (Table 4). Stratification by age revealed that heterogeneity was substantially reduced in certain age-based subgroups. Specifically, the $I^{2}$ dropped to 64.1% in the <75-year group at 12 months, and to 19.98% in the ≥75-year group at 6 months, suggesting that patient age may influence treatment response consistency (Table 5).

Subgroup analysis also showed that studies without co-treatment (i.e., TP monotherapy) and those targeting adjacent fractures tended to yield more consistent results favoring TP. We conducted a sensitivity analysis excluding studies involving adjacent fractures (Tseng and Su) [25,27]. The pooled mean differences in ΔVAS remained in favor of TP at both 6 months (−0.36; 95% CI: −0.47 to −0.24, *p* < 0.01) and 12 months (−0.44; 95% CI: −0.56 to −0.32, *p* < 0.01), despite persistent high heterogeneity ($I^{2}$ > 99%, *p* = 0.14).

In contrast, the study by Jung et al. [28], which involved denosumab co-administration with TP and showed a reversed treatment effect in favor of VP, contributed notably to the overall heterogeneity. The only RCT (Tseng et al.) [25] demonstrated consistent superiority of TP over VP across both pain relief and outcome precision. These patterns were stable across multiple time points, and were reinforced by sensitivity analyses.

## 4. Discussion

For patients suffering from OVCFs, the short-term goals of treatment are pain relief and recovery of quality of life, while the long-term goal is the prevention of new-onset fractures. Unfortunately, a significant portion of patients do not fully recover after an OVCF [8,29], even with active medical intervention. Ideal strategies should achieve both short-term (pain reduction, early restoration of quality of life) and long-term goals (fracture healing, progressive improvement of life quality, and prevention of new-onset OVCF) [26]. This meta-analysis identified the superiority of conservative treatment using TP. The well-known primary advantage of VP is pain control in the initial stages of fractures. However, there was no difference between TP and VP in the initial comparison within three months of fracture. In contrast, at the 6-month and 12-month points, the VAS score was significantly lower in the TP group.

Previous studies have reported that the minimal clinically important difference (MCID) for VAS in patients with lower back pain ranges from approximately 1.2 to 2.0 points [30,31,32,33]. In our analysis, the VAS reduction in the TP group was 1.65 points at 6 months and 1.50 points at 12 months compared to in the VP group, which meets or exceeds the lower threshold of MCID. These findings suggest that TP treatment not only yields a statistically significant improvement, but also confers a clinically meaningful benefit in pain relief. This is thought to be due to the significantly lower incidence of new-onset subsequent OVCFs and higher BMD in the TP group based on the rapid bone-healing effect of TP [17,18,19,20]. Based on these findings, we propose the use of TP in conservative treatment as a novel therapy for addressing acute OVCFs.

For patients with acute OVCFs and persistent pain, VP is effective and safe. Pain relief after VP is immediate, and is significantly greater than that achieved with conventional conservative treatment [34]. In this study, a subgroup meta-analysis limited to the immediate period within one week also showed that VP significantly outperformed in reducing VAS scores. It is commonly accepted that pain reduction following VP and kyphoplasty occurs due to the stabilization of the fractured vertebrae and the cessation of both microscopic and macroscopic movements at the site of the fracture [35]. Additionally, suggested mechanisms for alleviating pain involve the chemical neurolytic properties of Poly Methyl Methacrylate (PMMA) and the heat-induced neurolytic effects caused by the exothermic reaction of PMMA [35].

However, several adverse effects have been associated with vertebral augmentation, including cement leakage, osteonecrosis, recompression, infection, and adjacent-segment fractures [1,28,36]. The complications of cement leakage include neurologic deficits and pulmonary embolisms. Moreover, the incidence of new OVCFs after VP has been reported to range from 8% to 52% [37], with 41–69% of new vertebral compression fractures being immediately adjacent to the treated vertebra [1,38,39]. The relative risk of adjacent-level fracture is 4.62 times that of nonadjacent-level fracture [40]. In this study too, the odds proportion of new-onset OVCFs in the VP group was significantly higher (0.15 with 95% CI, 0.04–0.51, *p* < 0.01).

The issue of inhibited physiologic bone healing following VP is also a concern. PMMA-based bone cement is not ideal for bone ingrowth due to its biologically inert characteristic, meaning it does not degrade [41,42]. After VP, a peripheral halo was observed in over 10% of cases, and there was a higher re-fracture rate in the index vertebrae where VP was performed [43]. This suggests that the space needed for bone healing or regeneration is occupied by PMMA, hindering the process of natural bone healing [26].

Treating OVCF requires attention to both pain relief and the prevention of additional fractures. Failure in pain management due to a new-onset OVCF is a significant issue. Failure of fracture healing in OVCFs leads to intractable back pain associated with non-union [44]. Attention must be paid to treating both local fractures and overall low bone density. The addition of postural rehabilitation may also be beneficial [45]. In the present meta-analysis, TP achieved a greater improvement in BMD with statistical significance, with the overall mean difference estimated as 2.73 (95% CI, 0.99–4.48, *p* < 0.01). Since VP does not contribute to the prevention of further fractures, considering conservative treatment using bone anabolic agents as an alternative treatment option is viable.

TP has been demonstrated to promote bone healing and prevent fragility fractures in both rats and humans [46,47]. Although it has numerous clinical advantages, TP has some less-well-known contraindications, such as a history of radiation therapy and the presence of primary malignant and metastatic bone tumors [20]. And, TP includes higher costs compared to other conventional anti-osteoporosis treatments, it has a limitation of 24 months of use in a lifetime, and the inconvenience of daily injections may lead to decreased patient compliance, which is an essential consideration in the overall effectiveness of the treatment.

TP, as a bone anabolic agent, has shown superior therapeutic effects in various comparative studies with conventional anti-resorptive agents (bisphosphonates, BPs) [19,44,48]. BPs are commonly utilized for osteoporosis management. In the four studies on BPs included in this meta-analysis, BP was administered as a standard treatment to patients in the VP group. Clinical trial findings indicate a 40% to 50% reduction in the likelihood of OVCFs following three years of BP treatment [49,50]. Nonetheless, these agents might hinder the fracture-healing process by excessively suppressing physiologic bone turnover [51]. Furthermore, there is a noted association between the occurrence of intravertebral clefts and BP medication history [52]. In the treatment of acute OVCF patients with severe osteoporosis, the therapeutic effect of BP is too slow, and the use of these agents is associated with a high risk of new-onset fractures [25]. In a retrospective study of a total of 98 patients undergoing non-operative treatment for recent single-level OVCFs, the union rate at six months after treatment was 89% in the TP group and 68% in the BP group. Fracture-site surgical interventions were not required in the TP group; however, two patients in the BP group eventually underwent surgical treatment for symptomatic non-union or vertebral collapse. In addition, TP significantly affected union in cases of vertebral deformity progression [44]. Thus, the use of BP in conservative treatment does not effectively improve the treatment outcomes for acute OVCFs. It could be inferred that employing a bone anabolic agent for active fracture healing may provide practical assistance.

In addition, TP seems to aid in the relief of acute pain. In this meta-analysis, there no significant difference was found between the VP group and the TP group in VAS scores over 3 months. However, during this period, the point estimates shift in a direction favoring TP as time progresses. This aligns with recent animal studies that reported that the time point for pain reduction with TP was earlier than that noted for its bone anabolic effects. The DRG neurons are a direct target of TP, and TP immediately induces an antinociceptive effect [53]. And, its inhibitory effect on inflammatory cytokine expression might be related to one of the mechanisms by which TP rapidly reduces pain [21].

Meanwhile, once vertebral necrosis occurs, achieving union becomes difficult in the presence of a vertebral cleft. When using TP, peripheral bridging of bones along the vertebral edges occurs, restoring the stability of the collapsed vertebra [44]. In relation to peripheral bridging around fractures, TP promotes ossification of the spinal ligaments and accelerates hyperplastic bone formation around vertebral fractures in cases of diffuse idiopathic skeletal hyperostosis [54,55].

On the other hand, a meta-analysis regarding radiologic outcomes related to sagittal alignment was also conducted, and the VP group showed a slight superiority in the increase in KA and AH loss or compression rate at 12 months, without statistical significance. Although the follow-up period was limited to three months, and thus it was not included in this meta-analysis, there was no difference in AH and posterior height between TP and VP in comparisons up to three months [26]. In studies involving patients who did not undergo VP, there was a statistically significantly lower compression rate in patients treated with TP [56]. In studies with patients who underwent VP, although not statistically significant, there was a smaller increase in KA and compression rate in cases where TP was used [57]. Since these two studies were not focused on a direct comparison between VP and TP, they were excluded from this meta-analysis.

This meta-analysis has several limitations. Firstly, the number of included studies was few. However, the present study is the first meta-analysis to offer a direct overview of conservative treatment using TP and VP comparison. Second, most of the included studies were non-RCTs, which may introduce selection bias and limit the strength of causal inference. Third, there was no standardization among the included studies regarding the basic strategies of conservative treatment and VP, including the bedrest duration, analgesic medication, combination with anti-resorptive drugs, method of VP or kyphoplasty, and time from injury to first treatment. Therefore, caution is necessary while interpreting our pooled estimates. Fourth, the CIs in some categories were too widely ranged to achieve precision or accuracy. Fifth, significant heterogeneity in the VAS metric raises concerns about drawing definitive conclusions. High heterogeneity across studies is a well-recognized challenge in meta-analyses comparing therapeutic interventions in heterogeneous populations. In our analysis, we observed considerable statistical heterogeneity in VAS outcomes at all time points. However, further exploration revealed that this heterogeneity could be at least partially explained by underlying clinical and methodological differences among the included studies. Age emerged as a key effect modifier. Younger patients (<75 years) consistently demonstrated greater and more homogenous pain relief with TP, while patients aged ≥75 years showed variable responses. The use of denosumab in the TP group in one study (Jung et al.) [28] likely introduced an additional confounding effect, as this was the only study that showed a treatment advantage for VP. Sensitivity analysis excluding this study resulted in an increased effect size and reduced heterogeneity, underscoring its impact on the pooled results. Furthermore, studies targeting adjacent fractures showed consistent benefits from TP, possibly due to its osteoanabolic effects in high-risk fracture zones. Lastly, the single RCT included (Tseng et al.) [25] provided robust support for TP’s effectiveness, adding methodological strength to the overall conclusions. The heterogeneity might also be due to the inconsistency in the prescription of opioids and nonsteroidal anti-inflammatory drugs. However, the recommendations for these drugs are all based on weak evidence and lack consistency across guidelines [3,58]. Taken together, these sources of clinical and methodological inconsistency contribute to lowering the overall level of evidence and weakening the strength of the meta-analysis. Lastly, our analysis exclusively focused on VP as the surgical intervention for comparison with conservative treatment. While this approach was chosen to ensure homogeneity within the surgical intervention group and to facilitate a direct 1:1 comparison with conservative treatment, it inherently excluded kyphoplasty, which is a widely performed cement augmentation technique in clinical practice. As a result, the findings may not fully represent the broader spectrum of outcomes associated with cement augmentation procedures. Future studies and updated meta-analyses should aim to include kyphoplasty and its synonyms in order to provide a more comprehensive evaluation of all relevant interventions and enhance the generalizability of the results.

## 5. Conclusions

The novel conservative treatment, primarily utilizing TP as a bone anabolic agent, showed no significant difference to VP in terms of early period pain control associated with acute OVCFs. With the 6-month and 12-month results indicating a reduction in the new onset of subsequent OVCFs and a superior pain-control effect, this treatment strategy could be suggested as an alternative approach for acute OVCFs. Further large-population-based RCTs are needed to evaluate the efficacy of conservative treatment of OVCFs using TP compared with VP.

## Figures and Tables

**Table 1:** Evidence table.

Study /Design	Inclusion Criteria/Definition of Acute OVCF	Demographics and Follow-Up Periods	Intervention and Drug Dosage	Outcome Evaluation and Time Points	Complications	Results
Tseng et al. (2012) [25] /RCT	New-onset painful adjacent OVCFs after VP /Severe pain + based on MRI exam	TP (*n* = 22) Age 70.55 ± 4.10 Gender (F:M) 20:2 Baseline BMD −3.45 ± 0.73 F/U 24.63 ± 3.48 months	TP (20 μg/d, 18 months) +Ca (1~1.5 g/d) +VitD (800~1000 IU/d)	VAS, JOA, BMD, new-onset OVCF At 1, 6, 12, 18 months	VP: 6 new-onset adjacent OVCFs; 1 major bone cement extravasation requiring decompressive laminectomy; 2 minor bone cement extravasations TP: 1 New-onset adjacent OVCF	Treatment of post-VP adjacent OVCFs with TP (no new VP) was more effective than that of repeated VPs combined with an anti-resorber
		VP (*n* = 22) Age 75.95 ± 6.28 Gender (F:M) 20:2 Baseline BMD −3.76 ± 0.71 F/U 25.05 ± 3.42 months	VP +Alendronate (70 mg/wk) or Raloxifene (60 mg/d) +Ca (1–1.5 g/d) +VitD (800–1000 IU/d)			
Ma et al. (2020) [26] /Prospective, non-randomized, real-world study	55–80 years old Female OVCF (BMD < −2.5) /Based on 3.0T MRI and severe lower back pain, especially when walking or changing position	TP (*n* = 30) Age 67.35 ± 6.49 BMD (g/cm^2^) 0.62 ± 0.14 Fracture time (days) 14.25 ± 12.6 F/U > 3 months	TP (20 μg/d) +Ca (0.6 g/d) +VitD (500 IU/d)	VAS, ODI, SF-36, BMD, VH, Medical cost At 1 week, 1 month, 3 months	No significant complications were observed in either treatment	Both treatments with TP and VP significantly and similarly improved patients’ health quality, with reduced visual analog and ODI scores at end of first and third months. VP was more effective in reducing pain at the early time point (1 week)
		VP (*n* = 30) Age 70.84 ± 6.60 BMD (g/cm^2^) 0.66 ± 0.11 Fracture time (days) 12.26 ± 9.96 F/U > 3 months	VP +Alendronate (70 mg/wk) +Ca (0.6 g/d) +VitD (500 IU/d)			
Su et al. (2013) [27] /Prospective cohort and retrospective comparative study	New-onset painful adjacent OVCF after VP /Based on MRI exam, when they had their first painful VCF	TP (*n* = 32) Age 77.94 ± 7.44 Gender (F:M) 29:3 Baseline BMD −3.81 ± 0.93 F/U > 22.56 ± 4.71 months	TP (20 μg/d) +Ca (1.0–1.5 g/d) +VitD (800–1000 IU/d)	VAS, BMD, New onset OVCF At 1, 6, 12, 18 months	VP: 6 new-onset adjacent OVCFs; 2 new-onset nonadjacent OVCFs; 1 major bone cement extravasation requiring decompressive laminectomy; 1 minor bone cement extravasation TP: 1 new-onset adjacent OVCF	Therapeutic effects of TP are better than those of combination of VP with an anti-resorptive agent in terms of fracture prevention, BMD change, and sustained pain relief
		VP (*n* = 33) Age 73.12 ± 7.49 Gender (F:M) 30:3 Baseline BMD −3.29 ± 0.74 F/U > 23.27 ± 5.13 months	VP +Alendronate (70 mg/wk) or Raloxifene (60 mg/d) +Calcitonin (200 IU/d) +Ca (1.0–1.5 g/d) +VitD (400–800 IU/d)			
Jung et al. (2021) [28] /Retrospective comparative study	>75 years old, BMD < −3.0 OVCF, /Definition of acute OVCF was not reported. Painful OVCF (VAS about 7, initial VAS 7.62 ± 0.78 for TP, and 7.82 ± 0.59 for VP)	TP (*n* = 41) Age 81.07 ± 4.92 Gender (F:M) 38:3 Baseline BMD –3.51 ± 0.42 F/U > 1 yr	TP +Denosumab Combination > 1 yr +Ca	VAS, BMD, KA, New onset OVCF HS, Days ambulation At 0 (post), 1, 12 months	VP: 6 adjacent OVCFs at 1 yr F/U TP: No significant complications were reported	TP combination treatment without VP did not show significant differences in either clinical or radiologic results compared to VP cases
		VP (*n* = 45) Age 83.07 ± 5.02 Gender (F:M) 40:5 Baseline BMD –3.62 ± 0.44 F/U > 1 yr	VP +Alendronate, Risendronate +Ca			
Gou et al. (2022) [10] /Retrospective comparative study	(i) 60–90 years old; (ii) acute OVCF from low-energy trauma (fall from standing height or less; (iii) severe back pain; (iv) osteoporosis: CT values of L4 ≤ 80 HU /Trauma < 2 wks, based on MRI exam	TP (*n* = 22) Age 76.18 ± 5.43 Gender (F:M) 15:7 Baseline L4 CT HU 63.95 ± 6.93 F/U 14.05 ± 1.13 months	TP (20 μg/d, >6 months) +Ca (1.2 g/d) +VitD (800 IU/d)	VAS, ODI, SF-36, BMD, KA, New onset OVCF At 1, 3, 6, 12 months	VP: 8 new-onset OVCFs TP: 0 new-onset OVCFs No serious adverse events occurred in either group	TP was better than VP in terms of increasing spinal BMD to promote OVCF healing, reduce new OVCFs, and improve back pain, physical ability, and health-related quality of life
		VP (*n* = 49) Age 73.63 ± 5.78 Gender (F:M) 35:14 Baseline L4 CT HU 67.17 ± 11.55 F/U 13.82 ± 1.20 months	VP +Ca (1.2 g/d) +VitD (800 IU/d)			

BMD, bone mineral density; Ca, calcium; F/U, follow-up; HS, hospital stay; HU, Hounsfield unit; JOA, Japanese orthopaedic association; KA, kyphotic angle; ODI, Oswestry disability index; OVCF, osteoporotic vertebral compression fracture; RCT, randomized controlled trial; SF-36, 36-item Short Form; TP, teriparatide (conservative treatment using teriparatide); VAS, visual analog scale; VH, vertebral height; VitD, vitamin D; VP, vertebroplasty.

**Table 2:** Risk of bias assessment summary.

Study	Assessment	Domain	Risk Level	Justification
Tseng et al. (2012) [25]	RoB-2 (RCT)	Bias due to deviations from intended interventions	Some concerns	Blinding status of participants and care providers was not explicitly stated.
		Bias in measurement of outcome	Some concerns	Subjective outcomes such as VAS were used, and assessor blinding was not clearly described.
		Bias in selection of reported results	Some concerns	No pre-registered protocol or statistical analysis plan was referenced.
		Overall risk of bias	Some concerns	Lack of clarity in blinding and selective reporting domains.
Ma et al. (2020) [26]	ROBINS-I (non-RCT)	Confounding	Moderate	No statistical adjustment for baseline variables; possible selection bias by patient choice.
Su et al. (2013) [27]		Confounding	Moderate	Post hoc comparison group with no baseline adjustment.
Su et al. (2013) [27]		Classification of interventions	Moderate	Retrospective group received variable anti-resorptives.
Su et al. (2013) [27]		Deviations from intended intervention	Serious	Variation in treatment length and agent changes.
Su et al. (2013) [27]		Outcome measurement	Moderate	No blinding; subjective outcomes prone to bias.
Jung et al. (2021) [28]		Outcome measurement	Moderate	Outcome assessors were not blinded.

**Table 3:** Measurement of bone mineral density.

Study	Detailed Measurement Methods	Remarks
Tseng et al. (2012) [25]	Anteroposterior and lateral lumbar spine radiographs, measured by DEXA At least two evaluable vertebrae in the lumbar spine region (L1–L4) Vertebrae with structural changes or artifacts were excluded Diagnoses were not made based on single vertebral bodies	Included in the analysis (Figure 4A)
Ma et al. (2020) [26]	Both lumbar and femoral neck BMD were measured using DEXA and recorded separately	Excluded due to the absence of 1-year follow-up data
Su et al. (2013) [27]	Anteroposterior and lateral lumbar spine radiographs, measured by DEXA At least two evaluable vertebrae in the lumbar spine region (L1-L4)	Included in the analysis (Figure 4A)
Jung et al. (2021) [28]	Average value of lumbar spine radiographs (L1 to L4.), measured by DEXA	Included in the analysis (Figure 4A)
Gou et al. (2022) [10]	The CT value of L4 quantified on sagittal CT images CT values of L4 ≤ 80 HU were applied to the osteoporosis diagnosis	CT values excluded due to incompatibility with DEXA T-scores

CT, computed tomography; DEXA, dual-energy x-ray absorptiometry; HU, Hounsfield unit.

**Table 4:** Summary of mean differences in pain reduction (ΔVAS) between teriparatide and vertebroplasty at 3, 6, and 12 months, with subgroup characteristics.

Study	Time point	MD	SE	CI Lower	CI Upper	Age	TP Drug	VP Drug	Fracture Type	RCT	Overall $I^{2}$ (%)
Ma et al. (2020) [26]	ΔVAS at 3M	0.55	0.10	0.36	0.74	<75	TP	BP	New	non	99.60
Jung et al. (2021) [28]	ΔVAS at 3M	0.74	0.03	0.67	0.81	≥75	TP+ Deno	BP	New	non	99.60
Gou et al. (2022) [10]	ΔVAS at 3M	−0.40	0.04	−0.48	−0.32	<75	TP	No	New	non	99.60
Tseng et al. (2012) [25]	ΔVAS at 6M	−1.15	0.10	−1.34	−0.96	<75	TP	BP	AdjFx	RCT	95.59
Su et al. (2013) [27]	ΔVAS at 6M	−1.96	0.10	−2.15	−1.77	≥75	TP	BP	AdjFx	non	95.59
Gou et al. (2022) [10]	ΔVAS at 6M	−1.84	0.05	−1.94	−1.74	<75	TP	No	New	non	95.59
Tseng et al. (2012) [25]	ΔVAS at 12M	−1.89	0.11	−2.10	−1.68	<75	TP	BP	AdjFx	RCT	99.84
Su et al. (2013) [27]	ΔVAS at 12M	−2.31	0.09	−2.49	−2.13	≥75	TP	BP	AdjFx	non	99.84
Jung et al. (2021) [28]	ΔVAS at 12M	0.22	0.03	0.15	0.29	≥75	TP+ Deno	BP	New	non	99.84
Gou et al. (2022) [10]	ΔVAS at 12M	−2.09	0.05	−2.19	−1.99	<75	TP	No	New	non	99.84

BP, bisphosphonate; CI, 95% confidence interval; Deno, Denosumab; MD, mean difference; RCT, randomized controlled trial; SD, standard error; TP, teriparatide; VAS, visual analog scale; VP, vertebroplasty.

**Table 5:** $I^{2}$ statistics by age-based subgroups for ΔVAS at each time point.

Time Point	Age Group	$I^{2}$ (%)	Study Count
ΔVAS at 3M	<75	98.81	2 [Ma et al. (2020), Gou et al. (2022)] [10,26]
ΔVAS at 3M	≥75	-	1 [Jung et al. (2021)] [28]
ΔVAS at 6M	<75	97.46	2 [Tseng et al. (2012), Gou et al. (2022)] [10,25]
ΔVAS at 6M	≥75	-	1 [Su et al. (2013)] [27]
ΔVAS at 12M	<75	64.07	2 [Tseng et al. (2012), Gou et al. (2022)] [10,25]
ΔVAS at 12M	≥75	99.84	2 [Su et al. (2013), Jung et al. (2021)] [27,28]

## Data Availability

The original contributions presented in this study are included in the article. Further inquiries can be directed to the corresponding author.

## Abbreviations

BPs = bisphosphonates, DEXA = dual-energy X-ray absorptiometry, KA = kyphotic angle, MCID = minimal clinically important difference, OVCF = osteoporotic vertebral compression fracture, RCT = randomized controlled trial, ROBINS-I = Risk Of Bias In Non-randomised Studies–of Interventions, RoB-2 = Risk of Bias for randomized trials, TP = teriparatide, VAS = visual analog scale, VP = vertebroplasty.

## References

[1] Oh Y., Lee B., Lee S., Kim J., Park J. (2019). Percutaneous Vertebroplasty versus Conservative Treatment Using a Transdermal Fentanyl Patch for Osteoporotic Vertebral Compression Fractures. J. Korean Neurosurg. Soc..

[2] Takahashi S., Inose H., Tamai K., Iwamae M., Terai H., Nakamura H. (2023). Risk of Revision After Vertebral Augmentation for Osteoporotic Vertebral Fracture: A Narrative Review. Neurospine.

[3] Lee S., Cho D.-C., Kim K.-T., Lee Y.-S. (2021). Evidence-based treatment of osteoporotic vertebral compression fracture. J. Korean Med. Assoc..

[4] Ameis A., Randhawa K., Yu H., Côté P., Haldeman S., Chou R., Hurwitz E.L., Nordin M., Wong J.J., Shearer H.M. (2018). The Global Spine Care Initiative: A review of reviews and recommendations for the non-invasive management of acute osteoporotic vertebral compression fracture pain in low- and middle-income communities. Eur. Spine J..

[5] Esses S.I., McGuire R., Jenkins J., Finkelstein J., Woodard E., Watters W.C., Goldberg M.J., Keith M., Turkelson C.M., Wies J.L. (2011). American Academy of Orthopaedic Surgeons clinical practice guideline on: The treatment of osteoporotic spinal compression fractures. J. Bone Jt. Surg. Am..

[6] Bae I.-S., Moon B.G., Kang H.I., Kim J.H., Jwa C., Kim D.R. (2022). Difference in the Cobb Angle Between Standing and Supine Position as a Prognostic Factor after Vertebral Augmentation in Osteoporotic Vertebral Compression Fractures. Neurospine.

[7] Giangregorio L.M., Macintyre N.J., Thabane L., Skidmore C.J., Papaioannou A. (2013). Exercise for improving outcomes after osteoporotic vertebral fracture. Cochrane Database Syst. Rev..

[8] Venmans A., Klazen C.A., Lohle P.N., Mali W.P., van Rooij W.J. (2012). Natural history of pain in patients with conservatively treated osteoporotic vertebral compression fractures: Results from VERTOS II. AJNR Am. J. Neuroradiol..

[9] Inose H., Kato T., Ichimura S., Nakamura H., Hoshino M., Takahashi S., Togawa D., Hirano T., Tokuhashi Y., Ohba T. (2022). Factors Contributing to Residual Low Back Pain after Osteoporotic Vertebral Fractures. J. Clin. Med..

[10] Gou P., Zhao Z., Yu C., Hou X., Gao G., Zhang T., Chang F. (2022). Efficacy of Recombinant Human Parathyroid Hormone versus Vertebral Augmentation Procedure on Patients with Acute Osteoporotic Vertebral Compression Fracture. Orthop. Surg..

[11] Yang E.Z., Xu J.G., Huang G.Z., Xiao W.Z., Liu X.K., Zeng B.F., Lian X.F. (2016). Percutaneous Vertebroplasty Versus Conservative Treatment in Aged Patients with Acute Osteoporotic Vertebral Compression Fractures: A Prospective Randomized Controlled Clinical Study. Spine.

[12] Klazen C.A.H., Lohle P.N.M., de Vries J., Jansen F.H., Tielbeek A.V., Blonk M.C., Venmans A., van Rooij W.J.J., Schoemaker M.C., Juttmann J.R. (2010). Vertebroplasty versus conservative treatment in acute osteoporotic vertebral compression fractures (Vertos II): An open-label randomised trial. Lancet.

[13] Firanescu C.E., de Vries J., Lodder P., Venmans A., Schoemaker M.C., Smeets A.J., Donga E., Juttmann J.R., Klazen C.A.H., Elgersma O.E.H. (2018). Vertebroplasty versus sham procedure for painful acute osteoporotic vertebral compression fractures (VERTOS IV): Randomised sham controlled clinical trial. BMJ.

[14] Kallmes D.F., Comstock B.A., Heagerty P.J., Turner J.A., Wilson D.J., Diamond T.H., Edwards R., Gray L.A., Stout L., Owen S. (2009). A randomized trial of vertebroplasty for osteoporotic spinal fractures. N. Engl. J. Med..

[15] Buchbinder R., Osborne R.H., Ebeling P.R., Wark J.D., Mitchell P., Wriedt C., Graves S., Staples M.P., Murphy B. (2009). A randomized trial of vertebroplasty for painful osteoporotic vertebral fractures. N. Engl. J. Med..

[16] Buchbinder R., Johnston R.V., Rischin K.J., Homik J., Jones C.A., Golmohammadi K., Kallmes D.F. (2018). Percutaneous vertebroplasty for osteoporotic vertebral compression fracture. Cochrane Database Syst. Rev..

[17] Peichl P., Holzer L.A., Maier R., Holzer G. (2011). Parathyroid hormone 1-84 accelerates fracture-healing in pubic bones of elderly osteoporotic women. J. Bone Jt. Surg. Am..

[18] Aspenberg P., Genant H.K., Johansson T., Nino A.J., See K., Krohn K., García-Hernández P.A., Recknor C.P., Einhorn T.A., Dalsky G.P. (2010). Teriparatide for acceleration of fracture repair in humans: A prospective, randomized, double-blind study of 102 postmenopausal women with distal radial fractures. J. Bone Miner. Res..

[19] Tsuchie H., Miyakoshi N., Kasukawa Y., Nishi T., Abe H., Segawa T., Shimada Y. (2016). The effect of teriparatide to alleviate pain and to prevent vertebral collapse after fresh osteoporotic vertebral fracture. J. Bone Miner. Metab..

[20] Lee S., Seo Y.J., Choi J.Y., Che X., Kim H.J., Eum S.Y., Hong M.S., Lee S.K., Cho D.C. (2022). Effect of teriparatide on drug treatment of tuberculous spondylitis: An experimental study. Sci. Rep..

[21] Dohke T., Iba K., Hanaka M., Kanaya K., Okazaki S., Yamashita T. (2018). Teriparatide rapidly improves pain-like behavior in ovariectomized mice in association with the downregulation of inflammatory cytokine expression. J. Bone Miner. Metab..

[22] Page M.J., McKenzie J.E., Bossuyt P.M., Boutron I., Hoffmann T.C., Mulrow C.D., Shamseer L., Tetzlaff J.M., Akl E.A., Brennan S.E. (2021). The PRISMA 2020 statement: An updated guideline for reporting systematic reviews. BMJ.

[23] Sterne J.A., Hernán M.A., Reeves B.C., Savović J., Berkman N.D., Viswanathan M., Henry D., Altman D.G., Ansari M.T., Boutron I. (2016). ROBINS-I: A tool for assessing risk of bias in non-randomised studies of interventions. BMJ.

[24] Sterne J.A.C., Savović J., Page M.J., Elbers R.G., Blencowe N.S., Boutron I., Cates C.J., Cheng H.-Y., Corbett M.S., Eldridge S.M. (2019). RoB 2: A revised tool for assessing risk of bias in randomised trials. BMJ.

[25] Tseng Y.Y., Su C.H., Lui T.N., Yeh Y.S., Yeh S.H. (2012). Prospective comparison of the therapeutic effect of teriparatide with that of combined vertebroplasty with antiresorptive agents for the treatment of new-onset adjacent vertebral compression fracture after percutaneous vertebroplasty. Osteoporos. Int..

[26] Ma Y., Wu X., Xiao X., Ma Y., Feng L., Yan W., Chen J., Yang D. (2020). Effects of teriparatide versus percutaneous vertebroplasty on pain relief, quality of life and cost-effectiveness in postmenopausal females with acute osteoporotic vertebral compression fracture: A prospective cohort study. Bone.

[27] Su C.H., Tu P.H., Yang T.C., Tseng Y.Y. (2013). Comparison of the therapeutic effect of teriparatide with that of combined vertebroplasty with antiresorptive agents for the treatment of new-onset adjacent vertebral compression fracture after percutaneous vertebroplasty. J. Spinal Disord. Tech..

[28] Jung J.Y., Lee B.H., Lee J.Y., Jeon H.J., Cho B.M., Kim S.Y., Park S.H. (2021). Clinical and radiological outcomes of denosumab and teriparatide treatment in elderly patients with osteoporotic spinal compression fracture without vertebroplasty. J. Korean Soc. Geriatr. Neurosurg..

[29] Jung H.J., Park Y.-S., Seo H.-Y., Lee J.-C., An K.-C., Kim J.-H., Shin B.-J., Kang T.W., Park S.Y. (2017). Quality of Life in Patients with Osteoporotic Vertebral Compression Fractures. J. Bone Metab..

[30] Jacob K.C., Patel M.R., Collins A.P., Park G.J., Vanjani N.N., Prabhu M.C., Pawlowski H., Parsons A.W., Singh K. (2022). Meeting Patient Expectations and Achieving a Minimal Clinically Important Difference for Back Disability, Back Pain, and Leg Pain May Provide Predictive Utility for Achieving Patient Satisfaction Among Lumbar Decompression Patients. World Neurosurg..

[31] Copay A.G., Glassman S.D., Subach B.R., Berven S., Schuler T.C., Carreon L.Y. (2008). Minimum clinically important difference in lumbar spine surgery patients: A choice of methods using the Oswestry Disability Index, Medical Outcomes Study questionnaire Short Form 36, and pain scales. Spine J..

[32] Ostelo R.W., de Vet H.C. (2005). Clinically important outcomes in low back pain. Best Pract. Res. Clin. Rheumatol..

[33] Than C.A., Adra M., Curtis T.J., Shi A., Kim G.E., Nakanishi H., Matar R.H., Brown J.M.M., Dannawi Z., Beck B.R. (2023). The effect of exercise post vertebral augmentation in osteoporotic patients: A systematic review and meta-analysis. J. Orthop. Res..

[34] Ploeg W.T., Veldhuizen A.G., The B., Sietsma M.S. (2006). Percutaneous vertebroplasty as a treatment for osteoporotic vertebral compression fractures: A systematic review. Eur. Spine J..

[35] Shah R.V. (2012). Sacral kyphoplasty for the treatment of painful sacral insufficiency fractures and metastases. Spine J..

[36] Choi J.H., Kang H.D., Park J.H., Gu B.S., Jung S.K., Oh S.H. (2017). The Efficacy of Fentanyl Transdermal Patch as the First-Line Medicine for the Conservative Treatment of Osteoporotic Compression Fracture. Korean J. Neurotrauma.

[37] Zhang Z., Fan J., Ding Q., Wu M., Yin G. (2013). Risk factors for new osteoporotic vertebral compression fractures after vertebroplasty: A systematic review and meta-analysis. J. Spinal Disord. Tech..

[38] Kamano H., Hiwatashi A., Kobayashi N., Fuwa S., Takahashi O., Saida Y., Honda H., Numaguchi Y. (2011). New vertebral compression fractures after prophylactic vertebroplasty in osteoporotic patients. AJR Am. J. Roentgenol..

[39] Uppin A.A., Hirsch J.A., Centenera L.V., Pfiefer B.A., Pazianos A.G., Choi I.S. (2003). Occurrence of new vertebral body fracture after percutaneous vertebroplasty in patients with osteoporosis. Radiology.

[40] Trout A.T., Kallmes D.F., Kaufmann T.J. (2006). New fractures after vertebroplasty: Adjacent fractures occur significantly sooner. AJNR Am. J. Neuroradiol..

[41] Ku K.-L., Wu Y.-S., Wang C.-Y., Hong D.-W., Chen Z.-X., Huang C.-A., Chu I.-M., Lai P.-L. (2019). Incorporation of surface-modified hydroxyapatite into poly (methyl methacrylate) to improve biological activity and bone ingrowth. R. Soc. Open Sci..

[42] Vaishya R., Chauhan M., Vaish A. (2013). Bone cement. J. Clin. Orthop. Trauma.

[43] Kim K., Kuh S., Park J., Kim K., Chin D., Cho Y. (2012). What is the importance of “halo” phenomenon around bone cement following vertebral augmentation for osteoporotic compression fracture?. Osteoporos. Int..

[44] Iwata A., Kanayama M., Oha F., Hashimoto T., Iwasaki N. (2017). Effect of teriparatide (rh-PTH 1-34) versus bisphosphonate on the healing of osteoporotic vertebral compression fracture: A retrospective comparative study. BMC Musculoskelet. Disord..

[45] Scaturro D., Rizzo S., Sanfilippo V., Giustino V., Messina G., Martines F., Falco V., Cuntrera D., Moretti A., Iolascon G. (2021). Effectiveness of Rehabilitative Intervention on Pain, Postural Balance, and Quality of Life in Women with Multiple Vertebral Fragility Fractures: A Prospective Cohort Study. J. Funct. Morphol. Kinesiol..

[46] Alkhiary Y.M., Gerstenfeld L.C., Krall E., Westmore M., Sato M., Mitlak B.H., Einhorn T.A. (2005). Enhancement of Experimental Fracture-Healing by Systemic Administration of Recombinant Human Parathyroid Hormone (PTH 1–34). JBJS.

[47] Neer R.M., Arnaud C.D., Zanchetta J.R., Prince R., Gaich G.A., Reginster J.-Y., Hodsman A.B., Eriksen E.F., Ish-Shalom S., Genant H.K. (2001). Effect of parathyroid hormone (1–34) on fractures and bone mineral density in postmenopausal women with osteoporosis. New Engl. J. Med..

[48] Min H.K., Ahn J.H., Ha K.Y., Kim Y.H., Kim S.I., Park H.Y., Rhyu K.W., Kim Y.Y., Oh I.S., Seo J.Y. (2019). Effects of anti-osteoporosis medications on radiological and clinical results after acute osteoporotic spinal fractures: A retrospective analysis of prospectively designed study. Osteoporos. Int..

[49] Black D.M., Cummings S.R., Karpf D.B., Cauley J.A., Thompson D.E., Nevitt M.C., Bauer D.C., Genant H.K., Haskell W.L., Marcus R. (1996). Randomised trial of effect of alendronate on risk of fracture in women with existing vertebral fractures. Fracture Intervention Trial Research Group. Lancet.

[50] Ettinger B., Black D.M., Mitlak B.H., Knickerbocker R.K., Nickelsen T., Genant H.K., Christiansen C., Delmas P.D., Zanchetta J.R., Stakkestad J. (1999). Reduction of vertebral fracture risk in postmenopausal women with osteoporosis treated with raloxifene: Results from a 3-year randomized clinical trial. Multiple Outcomes of Raloxifene Evaluation (MORE) Investigators. JAMA.

[51] Matos M.A., Tannuri U., Guarniero R. (2010). The effect of zoledronate during bone healing. J. Orthop. Traumatol..

[52] Ha K.Y., Park K.S., Kim S.I., Kim Y.H. (2016). Does bisphosphonate-based anti-osteoporosis medication affect osteoporotic spinal fracture healing?. Osteoporos. Int..

[53] Tanaka T., Takao-Kawabata R., Takakura A., Shimazu Y., Nakatsugawa M., Ito A., Lee J.W., Kawasaki K., Iimura T. (2020). Teriparatide relieves ovariectomy-induced hyperalgesia in rats, suggesting the involvement of functional regulation in primary sensory neurons by PTH-mediated signaling. Sci. Rep..

[54] Hamano H., Takahata M., Ota M., Hiratsuka S., Shimizu T., Kameda Y., Iwasaki N. (2016). Teriparatide Improves Trabecular Osteoporosis but Simultaneously Promotes Ankylosis of the Spine in the Twy Mouse Model for Diffuse Idiopathic Skeletal Hyperostosis. Calcif. Tissue Int..

[55] Matsumoto T., Ando M., Sasaki S. (2015). Effective treatment of delayed union of a lumbar vertebral fracture with daily administration of teriparatide in a patient with diffuse idiopathic skeletal hyperostosis. Eur. Spine J..

[56] Park J.H., Kang K.C., Shin D.E., Koh Y.G., Son J.S., Kim B.H. (2014). Preventive effects of conservative treatment with short-term teriparatide on the progression of vertebral body collapse after osteoporotic vertebral compression fracture. Osteoporos. Int..

[57] Kang J.H., Yang S.M., Im S.B., Jeong J.H. (2019). Can Three Months of Teriparatide Be One of Treatment Options for Osteoporotic Vertebral Compression Fracture Patients?. Korean J. Neurotrauma.

[58] Parreira P.C.S., Maher C.G., Megale R.Z., March L., Ferreira M.L. (2017). An overview of clinical guidelines for the management of vertebral compression fracture: A systematic review. Spine J..

